# The British NHS at 75: Past, Contemporary, and Future Challenges

**DOI:** 10.1177/27551938241280175

**Published:** 2024-10-07

**Authors:** Ben Williams

**Affiliations:** Department of Social Sciences, 6249 Edge Hill University, Ormskirk, UK

**Keywords:** NHS, Beveridge, austerity, welfare state, welfare reform, pandemic.

## Abstract

This article assesses how and why the National Health Service (NHS) has remained at the forefront of British politics and society for 75 years amid significant economic pressures, organizational strain, shifting ideological trends, ongoing reforms, and an unprecedented public health emergency. The postwar “years of consensus” evolved into alleged decline and ostensible neglect during the 1980s, while New Labour sought to rejuvenate this core public service after 1997, featuring investment and often controversial reforms that challenged the party's social democratic values. Amid the New Labour era, NHS powers filtered down to devolved administrations, while from 2008 retrenchment and austerity ensued, fueled by global recession. Austerity eventually subsided, yet from early 2020 the NHS swiftly faced the extreme conditions of the globalized Covid-19 pandemic. The service continues to face challenges regarding its longer-term viability, and this article analyzes this scenario, within the context of the NHS's 75-year historical legacy, its contemporary status, comparative international trends, and likely future evolution.

## Introduction, Historical Context, Theoretical Framework and Methodology

The National Health Service (NHS) remains a critical element of British governance in the twenty-first century, a high-profile social policy and a prominent component of the contemporary welfare state. Since its creation in 1948, the primary focus of the NHS has been delivering universalized public health needs, with its central ethos aligned with William Beveridge's original sentiments (in 1942) to provide quality health care “from the cradle to the grave,” directly funded from government taxation and providing unconditional treatment regardless of ability to pay. Indeed, the NHS's essential mission was concisely summarized by the cabinet minister who presided over its introduction, Aneurin Bevan, who declared in Parliament in 1946 that “A person ought to be able to receive medical and hospital help without being involved in financial anxiety”.^1^ This outlook derived from a blend of humanitarian obligations and socialist ideology and values, both of which shaped the original service model,^2^ and have been central to this policy sphere and associated political debate ever since. The NHS has subsequently been described as having “no parallel in terms of its resilience . . . longevity and its abiding appeal”^3^ among the wider population, and within this context, polling has consistently indicated that the NHS retains significant public popularity since its creation amid the collectivist mood in the aftermath of World War Two.

Former Conservative Chancellor of the Exchequer Nigel Lawson once notably referred to the NHS as “the closest thing the English have to a religion, with those who practice it regarding themselves as a priesthood”.^4^ Such wider reverence has been further strengthened in recent times by the impact of the COVID-19 pandemic and the critical role played by the NHS throughout it. Yet this prolonged episode also exposed the underlying and recurring longer-term challenges pertaining to the sheer size, complexity and demographic variables facing the NHS as an organization (and which was further complicated and recalibrated with the advent of post-1999 devolution). It also raised renewed questions as to whether such foundational socialist and collectivist principles can be sustained amid demographic change, rising costs, and global capitalist structures and dynamics while continuing to deliver its core services into the future,^5^ particularly in comparison to other western health care systems. Within the context of its vast size and expense, by the first decade of the twenty-first century the NHS had grown to employ approximately 1.4 million people with an annual cost of over £100 billion and rising (in England); which provides significant political challenges in terms of its effective functional operation in the early twenty-first century.

In theoretical terms, the article seeks to focus on the key research question of how practical and political experiences of the past can be re-embedded as important factors and dynamics in moulding the future evolution and direction of the NHS. This is in the particular context of ongoing tensions and debate between its original collectivist ethos, versus rival “reformist” and “neoliberal” proposals for how the service model should alternatively function and be organized. A secondary research focus is how pressures of demographic change (a growing and ageing population) have also been a catalyst and a driver for organizational change and reform. Furthermore, the article aims to utilize and develop such a theoretical framework that engages with and acknowledges existing academic contributions on the specific subject matter of the NHS. Prominent examples include Bivins and her analysis of the legacy that the collectivist war effort had on the formation of the NHS,^6^ Sheard, who similarly describes the service in contextual terms as a “creature of its time”,^7^ Gorsky, who refers to “lessons from history” that can be derived from the universalist nature of the NHS,^8^ while Webster has emphasized the inherent “political” aspects of the organization's development and existence.^9^ The article seeks to build on and interact with notable literature that addresses core historical and political aspects of the NHS debate, while in the process realigning academic output with the contemporary context, notably by addressing the impact of austerity, key twenty-first century reforms, as well as the COVID-19 pandemic on the service.

Within this theoretical framework, it should be acknowledged that the NHS continues to offer an idealized model of a public health system, which many other countries have used as a template for emulation at various times, yet it has often struggled to deliver these ideals since its inception, and the reasons and factors for such problems and challenges of delivery will be analyzed. The article therefore seeks to assess and explain such key variables in NHS performance, which can in turn help to generate a more rigorous understanding of both past experiences and future prospects faced by the service model. This analysis would also seek to clarify the extent of the ongoing relevance and value of the NHS as a model for influencing other international health care systems, as well as to acknowledge and address recurring critiques from the political right.

Within the article's theoretical framework, the structure is broken down into five specified chronological phases to allow for an organized assessment and a logical and focused understanding of how the service has evolved over such a relatively long timescale. This structure also allows for clearer potential comparative analysis in relation to specified and contemporaneous developments within other global health care systems, notably so in the context of the post-2008 economic crash and associated financial strains that have consequently occurred and impacted across global public services. The methodology of research has identified and utilized a range of specified primary speeches, parliamentary debate, and policy documentation, particularly from the early twenty-first century period of British politics, in order to enhance the contemporary academic and original value of the article, combined with a series of news sources. Such varied primary sources are combined with further relevant secondary academic texts and biographies (to consolidate those key texts highlighted above) but with a more contemporary political and ideological emphasis.

## Phase One: The NHS, Postwar Challenges, and end of Consensus

In the broader postwar era, different governments from across the United Kingdom's political spectrum have repeatedly competed for the trusted mantle of who can most effectively manage the NHS, with it regularly being an issue at the top of voters’ priorities at general election time. In the process, Salter suggests that the various parties and their political figureheads have been faced with addressing a “never-ending public clamour for improved state health services”.^3^^(p.4)^ This historic legacy has meant that the emergence and consequent management of the NHS has been intertwined with political pressures deriving from public opinion, principally because it is ultimately a public body, directly funded by the taxpayer, addressing never-ending public health demands. This has resulted in an ongoing battle between rival political parties and associated ideological values to provide appropriate social and welfare rights to a demanding, growing and ageing population (aligned with similar demographic trends in other western countries).

However, a fundamental problem with addressing such elements of demand, supply and demographic change is that NHS provision and delivery appears infinite and has experienced inexorable growth from the earliest phase of the service's existence.^10^ In subsequent years it has effectively been a victim of its own success, namely by creating the conditions for an ageing population that lives longer, but which requires greater usage of public health services as a consequence. In practical terms, the supply of this core public service has, therefore, always had clear practical and financial limits, creating a rationing and streamlining of resources,^11^ leading to limited patient choice and with aspects of the service often prioritized over others (e.g., Bevan's resignation over dental and optical charges in 1951, or more recently the recurring annual “winter crisis”). Such organizational dynamics and pressures have therefore been an ongoing and recurring theme, and Webster has described the NHS as functioning and regularly existing in an “atmosphere of crisis”,^9^ which has had significant implications for successive governments when formulating and implementing NHS policymaking.

Having been established by the reforming postwar Labour Party-led government under Clement Attlee, the NHS was a specific model of “statist” public health delivery that the Conservative Party opposition was critical and skeptical of, regardless of the party leadership's experiences within an explicitly statist wartime coalition. Indeed, during the initial NHS parliamentary debates between 1946 and 1948, various Conservative politicians dismissed it as an expensive proposal and a long-term burden on the taxpayer, seemingly a prolonged vestige of the bureaucratic governmental structure that had prevailed during World War Two. This opposition was visible in the party's MPs repeatedly voting against its introduction, a stance for which the principal architect of the NHS Bevan described them as being “lower than vermin”.^12^ Yet while the “One Nation” Conservative tradition swiftly and pragmatically accepted the expanded welfare state's postwar existence^13^ via their Industrial Charter (1947) and its interventionist ethos, critical voices have lingered on the political right ever since, dissenting from the postwar model of health service.

This has been evident in the emergence of bodies such as the free-market Institute of Economic Affairs (IEA) in 1955, which accelerated its negative analysis of the postwar welfare settlement during the economically turbulent 1970s in particular, and continues to question its cost and viability up to the present day.^14^ The resignation of Peter Thorneycroft as Conservative Chancellor of the Exchequer in 1958 (plus two junior ministers) over public spending increases also reflected this dissenting mood, which has seemingly stretched back some time. However, such ongoing criticisms from elements of the political right generated an often-negative public image for the Conservative Party regarding its evidential skepticism towards the “statist” model for the NHS and associated provision of broader welfare policy.

Such specific historical context has often meant that NHS policy has generally “been regarded as a core Labour issue . . . especially so since the 1980s”,^15^^(p. 77)^, which was a polarizing decade whereby the service came under renewed pressure from the Thatcher government's controversial reforms aligned with the prevailing mood of neoliberal marketization.^16^ This period witnessed the unravelling of three decades of the “post-war consensus”^17,18^ and the Labour Party subsequently warned of threats to the fundamental aims and original structural model of the NHS from the dominant Thatcherite “New Right” ideology^19^ that entailed relative fiscal retrenchment. As the 1980s progressed, there consequently emerged growing concerns about investment in the service, featuring perceptions of the NHS as underfunded and ill-equipped to meet patients’ expectations amidst a distinctive and emerging “consumerist” culture that fueled an even more demanding model of public service provision.

Such concerns about the future of the service and how it could best meet the demands of its users, or “consumers,” could be aligned with the contemporary New Right ideological narrative previously alluded to. This reformist agenda of service delivery gathered further momentum with the advent of the “internal market” (following the 1990 NHS and Community Care Act), which saw enhanced private elements introduced to some NHS services, heightening fears among Thatcher's critics that her ultimate aim was to “privatize” the service. Yet it has been noted by some observers that Thatcher was rather more pragmatic than ideological in this policy sphere, with no serious attempts “to dismantle the NHS, let alone privatise it”.^20^^(p. 93)^ However, this allegation of pursuing outright privatization was subject to much recurring conjecture, with her defenders dismissing such allegations as alarmist and unfounded. Nevertheless, Thatcher's political opponents persistently alleged she would have liked to have gone further in more radically reforming the NHS into a more streamlined model of service but was thwarted by public opinion due to the NHS being “rooted in popular affection”,^21^^(p. 212)^ hence why she “shied away from serious reform”.^22^^(p. 32)^ Yet in the post-1990 aftermath of Thatcherism, “opinion polls identified health care as one of the top issues for voters,”^15^^(p.77)^ with the Conservatives’ funding of key public and welfare services over almost two decades in office being increasingly viewed unfavorably.

The extent that the NHS was revalidated and reaffirmed as a key area of voter concern by the end of the twentieth century was perhaps best illustrated in the 1997 general election outcome, when increasing concerns about the Conservatives’ NHS record were contrasted with Labour's plans to “rebuild the NHS”.^23^^(p.98)^ Yet a more positive analysis of the NHS under Thatcher's premiership argues that it maintained integral NHS services and instigated reforms that have been claimed to be some of the administration's “most successful achievements” and that its internalized market reforms “actually strengthened the welfare state”.^21^^(p.397)^ This argument would instead suggest that Thatcher's administration successfully secured the longer-term survival of such core features as the NHS via its reformist outlook, as it ultimately acknowledged that the original postwar model of the NHS was unsustainable in the longer term.

## Phase Two: 1990s Onward: NHS Modernization and Reinvestment

Despite the concerted attempts to reform and instill greater efficiency into the service during the 1980s, the dilemma the Conservative Party faced was that voters became increasingly concerned about its intentions regarding NHS policy, according to contemporary polling,^24^ and this came to hamper the party's management of this policy area during its prolonged eighteen years in office up to 1997. This was despite Thatcher's successor, John Major, showing more genuine interest and commitment to NHS policy after 1990, which was reflected in increased public investment in the service.^25^ The Conservatives consequently experienced sustained exile from national governance between 1997 and 2010, and during this period the NHS continued to be a high-profile area of public policy during the longest ever period of continuous Labour government, consolidated as an issue of political strength for Labour as a critical social policy that impacted most, if not all, voters. The role of the NHS high up the political agenda was central to New Labour's electoral strategy from the mid-1990s onward; it was a core policy issue during the 1997 general election campaign, aligned with Tony Blair declaring emotively, and perhaps disproportionately, that there were “only 24 h to save the NHS” on the eve of his party sweeping to power.^26^ This approach and policy agenda subsequently resulted in a wave of record financial investment in the country's health service after 1997, with the rate of NHS spending steadily and comparatively accelerating the longer that Labour was in office, amid ambitious aims from approximately the year 2000 to bring “health spending in Britain up to the average of the European Union over five years”.^27^^(p.337)^ Continuing or recurring failings in the service were pointedly directed at the previous Conservative regime.

New Labour's approach to this policy sphere positively engaged with the electorate's contemporary mood, ostensibly and opportunistically harking back to the Attlee era and its NHS legacy, and established a narrative that contrasted the perceived underspending of the Conservative years in office with a relatively long period of steady growth in public spending on core public services such as health over the course of the Blair/Brown administrations after 1997. This was perhaps a harsh analysis of the Conservative record, given that NHS spending increased by an annual average of 3.2 percent in real terms during the Thatcher/Major periods of government. Nevertheless, historically high levels of public funds were made available for this critical area of social policy after 2002 in particular, when Chancellor Gordon Brown's initial fiscal “prudence” was gradually discarded.^28^ A prime reflection of this trend was the evidently growing and “particularly large average annual increases in spending on the NHS (5.7% a year)” between 1997 and 2010,^29^^(p.9)^ and such figures exposed Conservative vulnerabilities in both its NHS policy and broader approach to public services, while also aligning with a broadly supportive public mood toward such explicitly inflationary trends in government expenditure on public health.

NHS policy was therefore a critical component of New Labour's three successive general election victories between 1997 and 2005, although the Blair/Brown strategic approach to health policy was often ambiguously “triangulated” in its direction, aligned with the more pragmatic “Third Way”.^30^ This was because alongside significant additional spending, there was continuation with aspects of the Thatcherite consumerist reforms of the 1980s and 1990s. This flexible acceptance of some private influence within the NHS was evident in further private investment via the regular usage of PFI (essentially public–private funding partnerships), which was often criticized as representing poor value for money.^31^ In addition, Blair's New Labour instigated the extended autonomy of hospital trusts into controversial foundation hospitals, enhanced managerial responsibilities for doctors, and private (nonstate) providers delivering some NHS provision in a more flexible way; allegedly a form of privatization by “stealth”^32^ according to Shaw, among others. Such bipartisan policy continuation, aligned with the neoliberal elements of the 1980s, reflected a new NHS consensus (in terms of the evolving service model) whereby a “marketized” service and performance would continue despite the NHS being historically based on statist and “universal” principles. On another policy dimension, the inception of devolution from 1999 also saw a practical decentralization of the service's organizational structure.

This pragmatic Blairite approach acknowledged the realities of the consumerist health care environment that had been inherited, while being aligned with Blair's own pragmatic belief that people were primarily focused on functional outcomes and a “heath service that worked”,^27^^(p.330)^ rather than hung up on ideological dogma regarding its organization, structure, and funding. What was ultimately a more nuanced and less ideological policy direction was consistent with New Labour's potent tactic of “triangulation” and desire to outflank political opponents by adopting and transcending some of their most popular and effective policies. The subsequent embracing of aspects of choice and marketization within core public services enigmatically blurred New Labour's approach after 1997 and aligned elements of its own NHS agenda with features of both the Thatcher and Major governments in the process.^32^

Nevertheless, an untrustworthy Conservative image aligned with lingering Thatcherite ideology continued to persist negatively within wider public opinion, which was cited by voters and noted by various contemporary observers in the party's three consecutive electoral defeats between 1997 and 2005.^33,34,35^ Spending on the NHS tripled in real terms in a decade, and Blair consequently proclaimed that his administration had indeed “saved” it.^36^ While such developments appeared to condemn the previous Conservative period in office, such a scenario could nevertheless be seen as both an opportunity and a challenge from the perspective of Conservative Party modernizers, particularly so after the “modernizing” candidate, David Cameron, became party leader in 2005.

What entailed was a prolonged attempt to “detoxify” the Conservative Party's image regarding NHS policy (and broader social policy matters), particularly and urgently so in the aftermath of an unprecedented third successive general election defeat in 2005. Within this context, Cameron's Conservative Party explicitly acknowledged the need to consolidate Labour's public expenditure commitments in this policy area, marking a fundamental strategic policy shift from a Conservative perspective. These attempts were undermined when some Conservative politicians continued to criticize the NHS's statist principles and bureaucratic tendencies, but the “modernizing” Conservative hierarchy also acknowledged the relative unpopularity of the market-friendly “pro-private” approach to health policy. On this basis, the prominent 2005 general election policy “The Patient's Passport” that advocated state subsidies for private health care (seemingly too radical for the broader public mood) was swiftly discarded within the first months of Cameron's leadership in early 2006.

Cameron's concerted attention to this core social policy area from the outset of his leadership was notably evident when he stated in his first party conference speech as leader in the autumn of 2006 that “Tony Blair explained his priorities in three words: education, education, education, I can do it in three letters: NHS”.^37^ Cameron's personal connection to the issue derived from NHS treatment of his son's serious illness (epilepsy and cerebral palsy), which reenforced it as his “number one priority”^38^ if he became Prime Minister, while thematically advocating the “politics of the family”^39^ and proclaiming in a more socially-fused narrative that the creation of the service was “one of the greatest achievements of the twentieth century”.^40^ While opponents had often criticized the Conservative Party for opposing the establishment of the NHS in 1948, Cameron's “modernizers” emphasized that their party had in fact advocated their own distinct (and less bureaucratic) public health policy model in the 1940s,^41^ preferring a fused state/market model similar to much of postwar continental Europe, and had subsequently managed the NHS for a longer period in office than any other political party.

While pursuing what was claimed to be a more “compassionate” social policy agenda,^42^ Cameron nevertheless rejected the dominance of the centralized and bureaucratic state, pledging additional public funds that were conditional on reform, while advocating more devolved service provision across diverse nonstate providers, an approach which formed a key part of his “Big Society” narrative from 2009 onward.^43,44,45^ In pursuing this somewhat ambitious and dynamic agenda to reformulate the structure and management of the NHS, Cameron swiftly embraced a more pragmatic middle ground. This approach subsequently aligned the Conservatives much closer to Labour's position in the build-up to the 2010 general election, primarily in terms of broadly matching NHS spending commitments. In his party's manifesto, Cameron explicitly promised to “back the NHS . . . [and] increase health spending every year”,^46^ a pledge subsequently reenforced in the 2010 Coalition agreement with the Liberal Democrats, which committed “funding for the NHS [to] increase in real terms in each year of the Parliament”.^47^^(p.24)^

This was, however, a conspicuously incongruous position given the distinctively thematic Conservative manifesto focus on significant retrenchment and broader public spending cuts to tackle the national deficit. Compared to the pre-1997 era, Conservative NHS policy appeared more nuanced under Cameron, and particularly sought to transcend the New Right's primary economic legacy. However, there were some public expenditure modifications following the 2008 global economic crash, subsequently entailing cautious conditions being instilled alongside otherwise solid NHS funding promises. This was specifically underscored by the affirmation that the rate of Labour's expenditure increase would not continue under an incoming Conservative administration, and which acknowledged that the rate of NHS spending had been on an upward curve and required more prudent rates of growth in the aftermath of the 2008 slump. Indeed, Cameron observed that over two decades, NHS spending had more than doubled in real terms from £38bn to £103bn^48^; yet others would cite that NHS spending level remained comparatively low by various European standards during the first decade of the twenty-first century, namely in relation to France and Germany.

## Phase Three: The NHS and Coalition Government (2010–2015)

Having finally returned to government from 2010 in a coalition administration with the Liberal Democrats, Conservative ministers emphasized the party's much-heralded pre-election pledge of increased NHS investment, which broadly aligned with the more expansive health policy of the junior coalition partners. As previously highlighted, this prominent commitment represented a relatively consistent policy stance since 2005, which Prime Minister Cameron re-affirmed in mid-2011 by stating “We will not cut spending on the NHS, we will increase it”,^47^ with the NHS supposedly “ringfenced” from major cuts. Yet such spending pledges subsequently became subject to significant scrutiny, particularly given media claims that NHS spending levels were actually cut in real terms by £25 million in the financial year 2011–2012 when inflation was taken into account, leading to academic observations that “the NHS [was] not immune”^49^^(p.490)^ from the era of economic austerity, which was the emerging policy narrative that would dominate the next five years at least. In brief, NHS spending did increase overall in real terms after 2010, but at a slower rate than under the previous government.

Nevertheless, despite austerity's emerging prevalence, skepticism on the Conservate right continued regarding the party's initial acceptance of much of New Labour's social policy agenda and the apparent consensus that relatively high levels of public spending automatically equated to improved service levels between 1997 and 2010. Such sentiments were acknowledged by Health Secretary Andrew Lansley, who in 2010 remarked in a post-election TV interview that “Britain now spends European quantities of money [on the NHS] without achieving European standards of treatment”,^50^ which reflected a recurring Conservative criticism that Labour's post-1997 NHS investment had not always reached frontline services due to alleged bureaucratic diversions and obstructions.

This viewpoint influenced the Conservatives’ longstanding and historical critique of the increasingly centralized and statist approach of Labour's management of the NHS, and which influenced Cameron's emphasis that his post-2010 NHS investment program should be accompanied by a long-term “reforming” and de-bureaucratized outlook to “modernize the NHS—because changing the NHS today is the only way to protect the NHS for tomorrow”.^48^ Such comments emphasized the recurring Conservative belief that enhanced financial investment alone was not the “silver bullet” for maintaining a viable NHS in the longer term, while focusing on deficit reduction was consistent with the Big Society's emphasis of more streamlined and less bureaucratic public service delivery, which aspired towards “a significant reshaping of public services . . . empowering front-line staff . . . to get on with the job”.^44^^(p.216)^ This envisaged a more autonomous ethos with a clear reduction in centralized state control, perhaps symbolized in the NHS by the abolition of primary care trusts (PCTs) by 2013. However, an ongoing challenge was to keep a potentially skeptical public opinion on board amid such reforms and restructuring, alongside the large “client state” attached to the service, namely the trade unions and the vast NHS workforce, significant sectors of the electorate.

As the senior partners of the 2010–2015 coalition, the Conservative Party leadership aspired to approach the delivery of post-2010 NHS policy with an acute awareness of previous public trust issues regarding this policy sphere. Rising NHS costs had been particularly fueled for some time by the long-term demographic trends of a growing and ageing population, and figures supporting this analysis included a 61 percent projected increased in those aged over 65 in the United Kingdom by 2032, as well as the increased average life expectancy in the United Kingdom having risen by a substantial 30 years over the course of the twentieth century, which was also a broader western global trend.^51^ In many ways these demographic variables illustrated the NHS's postwar success in considerably prolonging average life expectancy via “improvements in health, diet and preventative care”,^52^ yet which had generated significant financial costs relating to NHS infrastructure and service viability in the long term. Such demographic patterns have been particularly identified by various commentators as a specific cause of an inexorable growth of welfare spending amidst the ongoing extension of social rights in the postwar era, a trend that has notably proved challenging for governments across different western countries during this historical period.

While adopting a more social-themed agenda that was distinctive from the more vigorously economic Thatcher ideology of the 1980s, the 2010–2015 Conservative-led coalition nevertheless sought to distinguish itself from the allegedly profligate fiscal tendencies and bureaucratic, centralizing instincts associated with the New Labour era. The ultimate outcome saw the emergence of a scenario whereby there was evidence of the coalition government's commitment to investment in such a key public service, while simultaneously attempting to restructure and reform it.^53^ As already alluded to, attempts to significantly reform the NHS had been a major policy dilemma faced by various administrations, notably the Thatcher government, and likewise during the New Labour period there were difficulties in reorganizing the service while also investing in it, particularly due to Labour's close links to public sector trade unions and the resistance this movement has often expressed towards public service reform (under governments of different parties). The historic parallels suggest that the proposed post-2010 reforms were therefore always likely to face significant political and economic challenges.

Beyond 2010, linked to these socioeconomic influences and structural organizational pressures were lingering ideological imperatives for policymaking, with Prime Minister Cameron affirming that “competition benefits patients”.^48^ This competitive edge to public service provision reflected further acceptance of aspects of the legacy of the 1980s. Andrew Lansley was influential in the high-profile commitment made by the Conservative-Liberal Democrat coalition that the 2010–2015 administration would aspire to “free NHS staff from political micromanagement . . . [and] stop the top-down reorganisations of the NHS”.^47^^(p.24)^ This pledge epitomized the coalition government's apparently distinctive emphasis on patient empowerment and the liberation of the citizen/consumer, while also ostensibly reducing “statist” NHS bureaucracy. So-called “top-down” reorganizations were identified as negatively prominent features of NHS policymaking associated with the previous Labour administration and were deemed as being undesirable from both a Conservative and indeed liberal (Liberal Democrat) perspective for the principal reason of financial cost and escalating organizational bureaucracy. Within this context, reducing NHS bureaucracy was identified as a key early priority in relation to a structural aspect of the unerring NHS monolith that Labour's thirteen years in office had seemingly failed to address. As a broader thematic issue, the longer term (postwar) upward spiral of cost and bureaucracy within the NHS saw spending on the service consequently increase ten times its original level (in real terms) since 1948, and specifically over five-fold during the past fifty years,^54^ although yet again, such inflationary tendencies were broadly in line with most other developed nations also.

Despite Cameron's claims to have “ringfenced” and maintained NHS spending regardless of the post-2010 austerity agenda, critical voices highlighted that practical policy implications amid cuts in overall government expenditure meant that the health department's budget was nevertheless placed under strain, rising by only 0.1 percent annually until 2014, compared to a 4.5 percent annual average for most of the service's lifetime since 1948.^55^ Such challenging spending levels were put into a stark and somewhat pessimistic historical context by NHS chief executive Sir David Nicholson, who stated that proposed post-2010 spending levels were “generous when you look across the rest of the public service. [But] there has never been a time where we have had four years of flat real growth. It is unprecedented”.^56^ This generated renewed fears of “rationing” and relative austerity within the service, raising further questions about the Conservative Party's relatively expansive pre-2010 NHS commitments.

The zenith of reformist NHS policy between 2010–2015 was the high-profile Health and Social Care Act (2012), applied to England only, which Andrew Lansley justified in terms of improving an existing service that would now be seen as

among the best in the world . . . where national standards and funding secure a high-quality, comprehensive service available to all, based on need and not the ability to pay; and where the power to deliver is in the hands of local doctors, nurses, health professionals and local communities.^57^

This legislation entailed significant service restructuring with enhanced focus on instilling greater competition and devolved provision via a wider range of NHS service providers, although as junior coalition partners the Liberal Democrats expressed skepticism (and it had not been part of the formal coalition agreement). However, they ultimately supported the general reforming thrust, and Deputy Prime Minister Nick Clegg offered reassurances that “If I felt it was privatising the NHS or tearing it limb from limb, it would never have seen the light of day”.^58^ This perhaps indicated that while sympathetic to more individual freedoms and less bureaucracy entailed by such reforms, any suggestions of a reversion to a neoliberal agenda reminiscent of the 1980s was blunted by the Liberal Democrats during the coalition's duration.

## Phase Four: Impact of Post-2010 NHS Reforms

This specific reformist legislation subsequently endured a prolonged and difficult passage through Parliament during 2011–2012, with significant amendments and concessions secured by Liberal Democrat peers in the House of Lords in particular, primarily regarding the proposed devolution of powers and general practitioner (GP) financial autonomy, alongside the scale of external private health care providers that emphasized a greater “choice” agenda. Despite such coalition dynamics and tensions, Andrew Lansley dismissed any overtly ideological agenda, arguing that “choice, competition and the involvement of the private sector should only ever be a means to improve services for patients, not ends in themselves”.^57^ Prime Minister Cameron and Deputy Prime Minister Clegg also aligned health reforms with their pragmatic (not ideological) instincts towards improving public service provision, namely that “a new approach to delivering public services is urgently needed [and] . . . signal[ing] a decisive end to the old-fashioned, top-down, take-what-you-are-given model of public services”.^59^^(p.6)^ Lansley was subsequently moved sideways from the Department of Health in autumn 2012 amid suggestions that he bore the brunt of significant opposition arising from the post-2010 NHS reforms, and linked to criticisms that such reforms were ideologically libertarian in nature, with the fallout threatening to further erode the Conservatives’ reputation in this key policy area.

Both before and during his premiership, David Cameron repeatedly voiced aspirations for “post-bureaucratic”^60^ NHS governance and broader public service delivery, with recurring comments about wasting too much money on “empty bureaucracy”^48^ rather than investing it on the frontline services. Yet despite such consistently reformist rhetoric, the warnings of persistent bureaucratic growth continued to be highlighted under his governance by libertarian critics and various think tanks, who expressed specific concerns about the apparent additional organizational bureaucracy created by Cameron's proposals to transfer NHS purchasing power to GPs, supposedly to save costs and restrict bureaucracy. Such ironic implications of the coalition government's specific NHS reforms were further consolidated by similar Labour criticisms that despite the much-publicized focus on reducing the hegemonic role of the state and aspirations to cut NHS bureaucracy by approximately a third,^61^ the practical implications of post-2010 NHS policy actually created further “statist” structural bureaucracy.

This specific criticism ultimately suggests that NHS policy direction during 2010–2015 instigated a top-down organizational restructuring despite coalition government promises to the contrary, creating further layers of associated bureaucracy and “red tape” that will be difficult to eliminate in ensuing years. This would vindicate Max Weber's sociological analysis^62^ from the early twentieth century, whereby society becomes more organizationally complex and bureaucratized as it evolves. Within this context, therefore, it appears that top-down bureaucracy is often an inevitable feature of a vast and complex public service such as the NHS, principally because its organizational management is heavily influenced by political pressures to meet inexorable public demands. On this premise, such attempts to reduce bureaucracy were frustrated by the reality of the NHS as a taxation-based economic system” which has traditionally been managed in a centralized, “top-down manner”.^3^^(p.11)^ This reformist approach therefore generated further bureaucratic pressures that were difficult to curtail, and this has been a recurring feature of NHS organizational cycles, with existing financial pressures recently exacerbated by the 2008 global financial crisis.

Such explicit reforms that focused on greater organizational efficiency and improved levels of performance created difficult practical implications, with polling figures indicating the lowest recorded health satisfaction surveys for thirty years,^63^ reflecting a 12 percent fall in public support between 2010–2011. This prompted critics to link the direction and focus of the NHS policy agenda since 2010 to a steadily worsening service, aligned with the austerity narrative. Prime Minister Cameron expressed awareness of rising opposition and criticism to his NHS reforms, declaring a willingness to “take a hit” (at least in the short term) from the wider public over the issue, but adding that the reforms were those of a “brave government”.^64^

## Phase Five: Post-Austerity NHS Policy

In the aftermath of such reforms and ongoing NHS spending commitments from Cameron to support a “seven-day NHS”, there followed the Conservatives’ somewhat unexpected 2015 general election victory, which was particularly so in the context of five years of austerity across the wider realm of public services amid often difficult coalition government tensions.^65^ However, the party's new majority status from 2015 saw its social and welfare policy agenda increasingly distracted by the imminent Brexit referendum, amid somewhat contentious Brexiteer claims that leaving the European Union could engineer a bonus of £350 million a week into NHS coffers. This reflected the ongoing importance of the NHS as a core policy issue for the electorate and was ultimately said to be one of the key factors that swayed many of those who supported Brexit.^66,67^ The promise of a post-Brexit financial windfall for the NHS has subsequently developed into one of the most contentious policy debates of subsequent years, with some skeptical observers questioning the veracity of such expansive funding pledges, regardless of the referendum's outcome seemingly indicating that a post-austerity future beckoned for the service.

NHS services had been progressively devolved and delivered to variable standards across the United Kingdom's national constituent parts since the late 1990s, with such devolved areas being autonomous in this policy sphere and (due to their anti-Conservative composition and control) not impacted by the various 2010–2015 “top-down” reforms. Nevertheless, ongoing concerns over the broader service's stability and future funding appeared to reach a crescendo at the 2017 general election amid ongoing frustration at the failure to practically deliver Brexit. Prime Minister Theresa May also performed an unconvincing mid-campaign U-turn on health and social care policy, while within this context Jeremy Corbyn's Labour opposition offered a far more expansive and ambitious spending program for the NHS and core public services.

Corbyn's approach was evidently aligned with conventional socialist ideology at the heart of the NHS's original agenda, which “struck a chord with the austerity weary electorate”^68^^(p.567)^ regarding the broader impact and delivery of public service provision. Consequently, both observers and frontline politicians noted a backlash against post-2010 austerity, whose “prolonged and relentless nature . . . played a part in the loss of (May's) parliamentary majority”.^69^^(p.19)^ Having therefore unexpectedly lost control of the House of Commons, a chastened Theresa May indicated at the Conservative Party's 2018 annual conference that the end of austerity loomed on the horizon once Brexit was implemented, as then scheduled for early 2019. This aspiration was initially evident in early 2018, with major implications for the NHS when May's government announced a new five-year funding settlement for the service in England, which equated to a “3.4% average real-terms increase . . . a significant improvement on the average 1% . . . since 2009/10”.^68^(p.84)

This dovetailed with a range of more expensive and interventionist “One Nation” style social policies that suggested May was notably keen to depart from the comparatively restrictive fiscal shackles of Cameron's domestic austerity agenda,^70^ although such aims ultimately never came to fruition due to the disruptive prevalence of the unerring Brexit saga and its global ramifications. May's successor Boris Johnson embraced the post-Brexit departure from the austerity agenda even more enthusiastically on becoming Prime Minister in mid-2019, with the NHS poised to reap further financial rewards in the process. From the outset, Johnson was more confident and vocal than May in utilizing “One Nation” language^71^ to bolster the post-austerity narrative, offering renewed momentum from this particular perspective, fully aware of likely electoral gains from such an approach while strategically linking further NHS investment to the apparent benefits of Brexit being finally delivered. The decisive abandonment of almost a decade of austerity politics came to fruition in the 2019 Conservative Party manifesto, which acknowledged that “spending cuts were a key weakness in 2017” and that significant extra spending pledges on key policy areas like the NHS could be viewed as an attempt to “distance himself from [the] unpopular austerity legacy . . . [and to] spend on the issues voters cared about”.^68^^(p.209)^

Despite such a populist approach to NHS policy-making that chimed with the views of “Red Wall”,^72^ pro-Brexit voters that Johnson (ultimately successfully) pitched his appeal to, during the 2019 campaign the Conservatives faced various allegations from Labour that the NHS was “up for sale” to international (namely American) commercial interests, ostensibly as part of an allegedly unfavorable post-Brexit trade deal with the United States. This could be linked to claims from various sources including the American Ambassador to the United Kingdom Woody Johnson and was a moment of potential danger for the Conservative electoral strategy within a policy area (as previously alluded to) that they were traditionally weaker on, and particularly as voter attention to the NHS “had been steadily rising as the campaign progressed”.^68^^(p.246)^ Although such allegations lingered beyond 2019, Johnson's disciplined campaign focus effectively neutralized such claims within the electoral arena and asserted there was no basis for them. Any immediate public backlash was subsequently limited, as evident in his eventually convincing 2019 electoral victory.

Indeed, a pivotal feature of this success was the Conservatives’ pledge of significant NHS investment, which appeared to transcend both Thatcherism and austerity. Johnson's electoral strategy regarding the NHS therefore outflanked Labour's recurring and perhaps predictable attacks on the issue, in recognition that the NHS remained a prominent policy of concern to voters, being the fourth most covered topic based on weekday press coverage.^68^^(p.377)^ Although the NHS continued to be criticized for not being historically funded to comparable levels of other western European states, the nature of the 2019 general election campaign and its outcome appeared to reaffirm that it remained a priority electoral issue, and that prominent manifesto commitments to invest in it was a key element of any vote-winning strategy.

The value and reverence that most British people continued to have for the NHS became even more apparent with the outbreak of the COVID-19 pandemic in early 2020—an unprecedented scenario that has been described as the NHS being placed on a “war footing” and akin to the “worst crisis since the Second World War”.^73^^(p. 239)^ Despite consequent acknowledgments that a global pandemic was always a (remote) prospect for any administration to deal with, Prime Minister Johnson could never have wholly envisaged facing one while making such bold NHS pledges while in electoral campaigning mode. As the crisis conditions of the pandemic evolved, major challenges emerged that struck at the viability of the overall health service, especially its service capacity issues, the delivery of the vaccine, insufficient staff personal protective equipment (PPE), and genuine fears that the NHS could be overwhelmed, amid allegations of underinvestment and not being prepared for such a scenario.^74^

Nevertheless, such enduring public affection for the NHS resulted in high-profile episodes such as the “clap for carers” campaign, which reflected widespread respect for the roles of medical staff, and which was a notable feature of the prolonged period of strain and crisis inflicted on the entire public health care structure throughout much of 2020 and early 2021. As an ostensible means of further strengthening the NHS going forward, the Johnson administration indicated plans to review some of the controversial Cameron-era NHS reforms and the ongoing bureaucracy that persisted. It also presided over a longer term and strategic “Five Year Forward View” from 2019, as well as instigating the 2022 Health and Care Act which sought to address inexorably rising service pressures deriving from the intensified interaction between health and social care provision.

## Conclusion: The NHS and The Future

As the 2020s proceed, the eightieth anniversary of the 1942 Beveridge Report has been notably commemorated as being the catalyst for the modern British welfare state,^75^ as has the 75th anniversary of the NHS. Many of the issues and challenges that were evident at the time of its inception continue to exist in the contemporary era, re-embedded as historical legacies, and have created ongoing challenges for this core public service. These include strains arising from infinite public demand, continuously rising financial costs stemming from a universalized ethos, the prioritization, provision and rationing of key services, ongoing bureaucracy, and optimizing the organizational structure. In addition, new and more complex pressures have emerged as the service has evolved, with the NHS largely responsible for creating the demographic conditions for a growing and ageing population that has subsequently put further strains on its functional performance, while as highlighted earlier, NHS functions have been devolved to the peripheral British regions from the late 1990s, marking a further significant evolution of the original service model.

While some broad cross-party ideological consensus remains about the value of the core Beveridge legacy, twenty-first century policy divergence has occurred in terms of the degree and extent of reform and modernization ostensibly required to maintain the NHS going forward. In observing its comparative effectiveness to other western health systems, reformers on both the mainstream political right and left both continue to demand further organizational changes and dynamism, entailing a more “marketized,” commercial, and conditional direction advocated from the right, while protecting the original universalized and statist model as prioritized by the traditional left. The crisis conditions of the COVID-19 pandemic and subsequent vaccine roll-out took NHS pressures to an unprecedented level, indicating just how critical, valued and enduringly popular the public service is in the contemporary era, although again highlighting the substantial, growing (and maybe even unsustainable) financial cost of the service.

Boris Johnson's ambitious pledges for “levelling up” in the context of his acquired “Red Wall” geographic domain placed further strain on NHS funding demands and associated infrastructure investment while exerting further internal ideological tensions upon his Conservative Party. Furthermore, dealing with the operational backlog created by the pandemic has emerged as a pivotal and demanding challenge for any current or future government.^76^ Nevertheless, the Conservatives’ refocused investment into the NHS and subsequent pandemic management sought to depoliticize and neutralize the electoral potency of the issue, blunting the impact of Labour's repeated attacks over NHS policy during the 2019 general election in particular, as the electoral outcome suggested. Indeed, by 2024 the Conservatives were boasting of delivering record levels of spending of £165.9 billion a year on the service in England^77^ (an apparent 40% increase since 2010). However, in the post-pandemic period Labour has reformulated its criticisms, arguing that the NHS was considerably underfunded when the COVID-19 health crisis erupted in early 2020, while a Conservative Member of Parliament (MP) defected to Labour in 2024 ostensibly over ongoing NHS policy concerns amid historically high waiting lists, which by 2023 had reached over seven million patients and continued to rise.^78^ Such issues evidently impacted the outcome of the 2024 general election, which the Conservatives lost badly, but with the incoming Labour administration consequently having to provide appropriate solutions in the immediate term.

This politically fluid context vindicates why over the course of the early twenty-first century, successive governments of various political complexions have sought to both invest in and reform the NHS in order to ostensibly secure its future and viability in the longer term, seeking electoral rewards accordingly. Indeed, while a degree of consensus remains as to the inherent value and utility of the NHS as a core public service, policy differences remain regarding various longer term organizational and structural challenges that it faces, often becoming more visible when various specific crises or problems erupt. Furthermore, the pressures of demographic change (also a global feature of health care provision) arguably demand more innovative policy responses compared to the consensual, early postwar era. This approach to both reform and investment can stem from either a pragmatic or an ideological imperative, with a prevailing acceptance since the 1980s of the requirement for a degree of private or marketized aspects to secure effective service delivery, yet while never wholly abandoning the ostensibly enduring values of the postwar Beveridge legacy. Indeed, Gorsky has positively argued for the organization's ability to embrace evolutionary reform, stating that the NHS “has repeatedly demonstrated capacity for innovation within a statist system and allayed uninformed prejudice against ‘socialised medicine,’” which can, aligned with Bevan's founding civilizational vision, make society “more wholesome, more serene, and spiritually healthier, if its citizens have the knowledge that they and their fellows, have access, when ill, to the best that medical skill can provide”.^8^^(p.58)^

While the political right has broadly tended to appear more enthusiastic for NHS reform in terms of critiquing the “years of consensus” from the Thatcherite and “small state” perspective, the ten-year Labour administration of Blair (1997–2007) in particular also pro-actively engaged with fairly significant reform (although with relatively greater financial investment). This suggests aspects of cross-party consensus about the necessity of organizational and structural reform, which in the modern political era has incorporated an evidently ideological Conservative catalyst and a more pragmatic Labour response, although on the more partisan fringes there remain both free marketeer and state socialist ideologues with very different and more radical proposed remedies. NHS policymaking has therefore been shaped by both historical circumstances and specific ideological policy responses to contemporary events, and such dynamics have helped to drive the service forward and maintain its ongoing existence. During the specified chronological phases within this article, such proposed initiatives, policies, and reforms have often faced practical, institutional, and public obstacles in the pursuit of maintaining and improving such public service provision, particularly when challenging the existing status quo. We can therefore conclude that such historical legacies have certainly helped to shape the system as it is today, but also acknowledge that such an ongoing cycle of further proposals and dynamics for NHS reform and innovation will inevitably continue, triggering further policy debate about the nature, durability, and practical purpose of the NHS for the foreseeable future.
